# Supplementary material for: PDE3A and GSK3B as Atrial Fibrillation Susceptibility Genes in the Chinese Population via Bioinformatics and Genome-Wide Association Analysis
Source: Biomedicines. 2023 Mar 15;11(3):908. doi: 10.3390/biomedicines11030908 (PMC10046458; doi:10.3390/biomedicines11030908)
Supplement: Supplementary file 1 [file biomedicines-11-00908-s001.zip › biomedicines-2230845-supplementary.pdf]

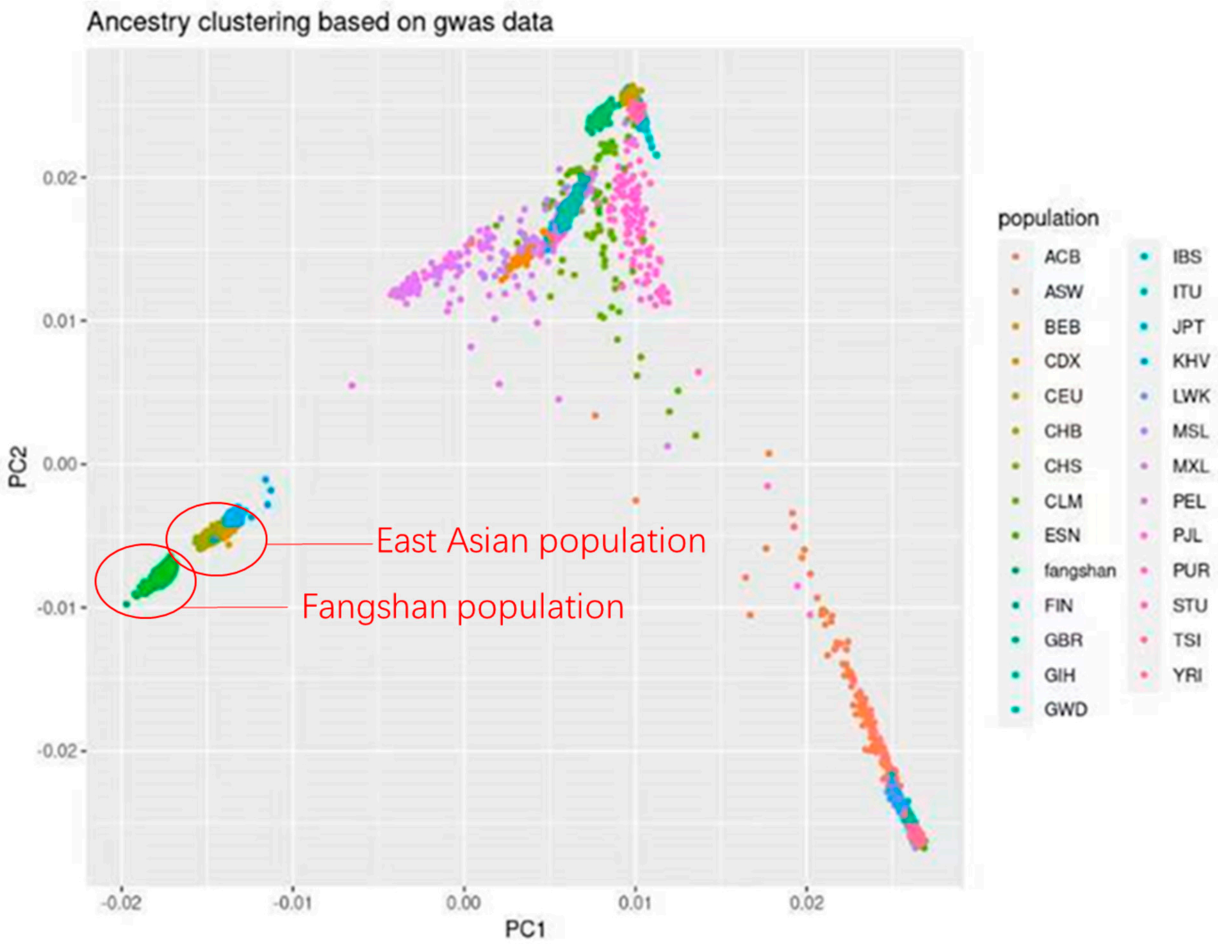

Figure S1. Principal component analysis of Fangshan population and various ethnic groups. The figure shows the first two principal components to reveal population structure. The distribution of PCAs in Fangshan population and East Asian population is basically the same.

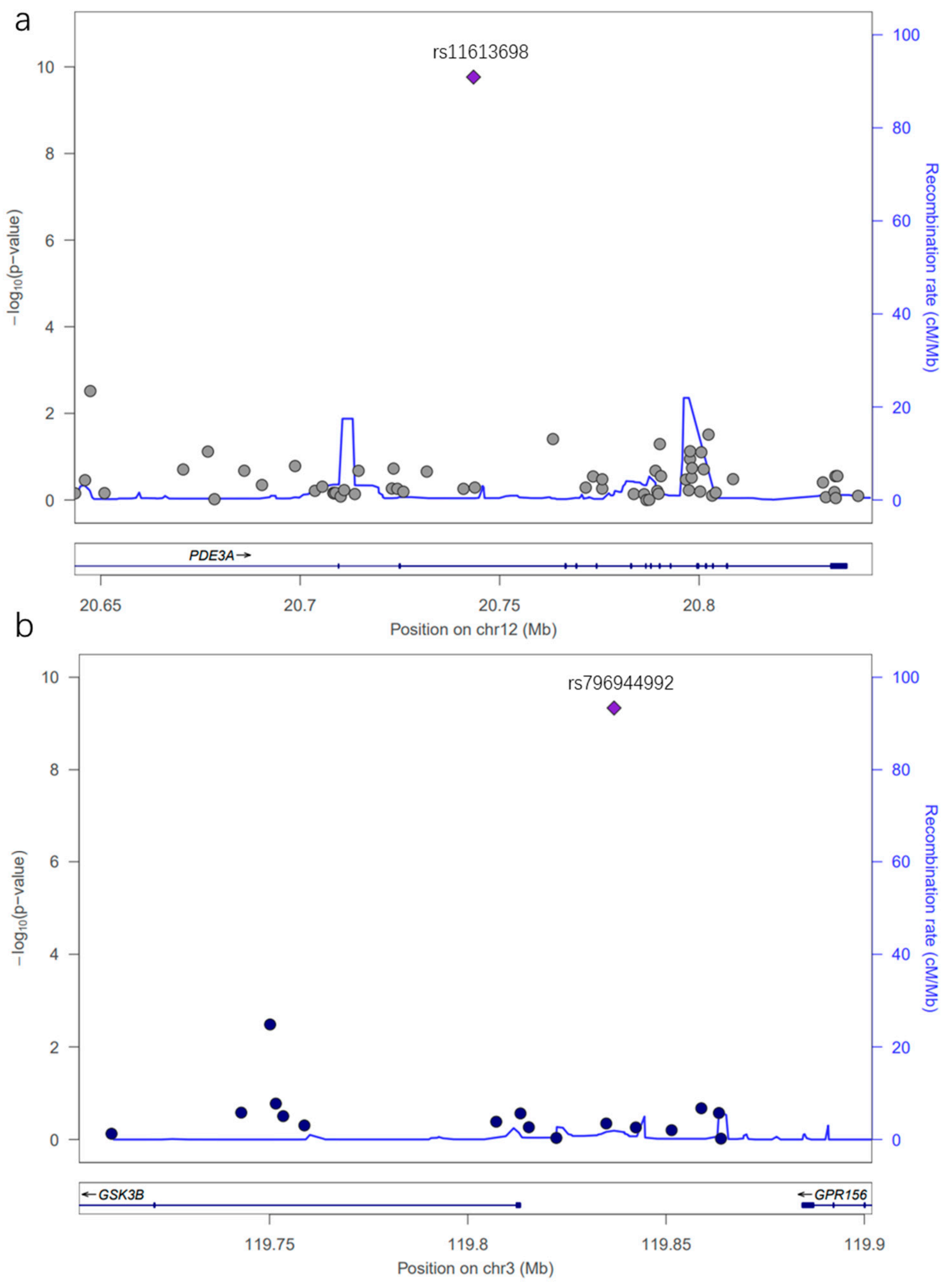

Figure S2. LocusZoom plots PDE3A rs11613698 and GSK3B rs796944992. SNPs are colored based on their correlation (r 2) with the labeled top SNP. Arrows on the horizontal blue lines show the direction of transcription.
